# Anti-Infective and Antiproliferative Potential of African Medicinal Plants

**DOI:** 10.1155/2012/535219

**Published:** 2012-06-21

**Authors:** Victor Kuete, Namrita Lall, Thomas Efferth

**Affiliations:** ^1^Department of Biochemistry, Faculty of Science, University of Dschang, P.O. Box 67, Dschang, Cameroon; ^2^Department of Plant Science, Faculty of Agricultural and Biological Science, Pretoria 0002, South Africa; ^3^Department of Pharmaceutical Biology, Institute of Pharmacy and Biochemistry, University of Mainz, Staudinger weg 5, 55128 Mainz, Germany

The importance of traditional medicine as a source of primary health care was first officially recognized by the World Health Organization (WHO) in the primary Health Care Declaration of Alma Ata (1978) and has been globally addressed since 1976 by the Traditional Medicine Programme of the WHO. In Africa, traditional healers and remedies made from plants play an important role in the health of millions of people. Scientific evidence of their pharmacological potential is being provided continuously. For this special issue, we have invited investigators to contribute original research articles as well as review articles that will provide evidences on the basis of traditional knowledge of the use of African medicinal plants used in the treatment of ailments. 

Six papers of this special issue focused on antimicrobial and two on antiproliferative properties of African medicinal plants. One paper of the issue reports the antibacterial properties of the methanol extracts of some Cameroonian medicinal plants and the effect of their associations with currently used antibiotics on multidrug-resistant (MDR) Gram-negative bacteria overexpressing active efflux pumps. Three extracts *Garcinia lucida, Garcinia kola,* and *Picralima nitida* showed significant activities against such bacteria and are suggested as possible alternative in chemotherapy involving MDR bacterial species. Another paper highlights the importance of *Annona senegalensis* and it active constituent, kaurenoic acid as a potential antibacterial  drugs  against  *Bacillus* *subtilis*,   *Pseudomonas* *aeruginosa*,   and *Staphylococcus aureus.* Another paper provides evidence on the inhibitory potential of the acetone and aqueous extracts of mature stem bark of *Sclerocarya birrea *on a panel of bacteria and fungi such *Streptococcus pyogenes, Plesiomonas shigelloides*, *Aeromonas hydrophila, Salmonella typhimurium, Cryptococcus neoformans, Candida glabrata, Trichosporon mucoides, *and *Candida krusei.* There is also a paper which demonstrates that extracts from South African medicinal plants *Euclea natalensis *A.DC., *Knowltonia vesicatoria * (L.f) Sims and *Pelargonium sidoides *DC can successfully be combined with isoniazid (INH) to improve it antimycobacterial activity. This paper also demonstrates that stigmasta-5,23-dien-3-ol isolated from *K. vesicatoria *is a bioactive compound against *M. tuberculosis*. Another paper reports the antimicrobial activity of the ethanol extract of *Artemisia afra *and identified some of its components acacetin, scopoletin and betulinic acid as bioactive compounds against Gram-positive bacteria (*Actinomyces naeslundii, Actinomyces israelii *and *Streptococcus mutans*), Gram-negative bacteria (*Prevotella intermedia, Porphyromonas gingivalis, *and *Aggregatibacter actinomycetemcomitans*) and a yeast, *Candida albicans*. One of these six papers provides information on the possible use of *Pterygota macrocarpa *and *Cola gigantean *in the fight of microbial infections involving *E. coli*, *P. aeruginosa*,*S. aureus*,*B. subtilis, *and *C. albicans*. 

Two papers of this special issue were related to cancer. Paper one provides information on the ethanol leaf extracts of *Harpephyllum caffrum *as an antityrosinase agent for dermatological disorders such as age spots and melasoma. The other paper reports the anticancer activities of African spices,*Origanum vulgare, Rosmarinus officinalis, Lavandula spica, Laurus nobilis, Thymus vulgaris, Lavandula-x-intermedia, Petroselinum crispum, Foeniculum vulgare, *and *Capsicum annuum L. (Paprika).* It identifies *L. nobilis *and *R. officinalis *as strong antiproliferative agents against the human cervical cancer cell line, HeLa.

*Victor Kuete* *Victor Kuete* *Namrita Lall* *Namrita Lall* *Thomas Efferth* *Thomas Efferth*

